# Lactoferrin in a Context of Inflammation-Induced Pathology

**DOI:** 10.3389/fimmu.2017.01438

**Published:** 2017-11-06

**Authors:** Marian L. Kruzel, Michal Zimecki, Jeffrey K. Actor

**Affiliations:** ^1^McGovern Medical School, University of Texas, Health Science Center, Houston, TX, United States; ^2^Polish Academy of Sciences, Institute of Immunology and Experimental Therapy, Wrocław, Poland

**Keywords:** lactoferrin, immunopathology, immunotherapy, inflammation, pathology

## Abstract

Much progress has been achieved to elucidate the function of lactoferrin (LTF), an iron-binding glycoprotein, in the milieu of immune functionality. This review represents a unique examination of LTF toward its importance in physiologic homeostasis as related to development of disease-associated pathology. The immunomodulatory nature of this protein derives from its unique ability to “sense” the immune activation status of an organism and act accordingly. Underlying mechanisms are proposed whereby LTF controls disease states, thereby pinpointing regions of entry for LTF in maintenance of various physiological pathways to limit the magnitude of tissue damage. LTF is examined as a first line mediator in immune defense and response to pathogenic and non-pathogenic injury, as well as a molecule critical for control of oxidative cell function. Mechanisms of interaction of LTF with its receptors are examined, with a focus on protective effects *via* regulation of enzyme activities and reactive oxygen species production, immune deviation, and prevention of cell apoptosis. Indeed, LTF serves as a critical control point in physiologic homeostasis, functioning as a sensor of immunological performance related to pathology. Specific mediation of tissue pathophysiology is described for maintenance of intestinal integrity during endotoxemia, elicited airway inflammation due to allergens, and pulmonary damage during tuberculosis. Finally, the role of LTF to alter differentiation of adaptive immune function is examined, with specific recognition of its utility as a vaccine adjuvant to control subsequent lymphocytic reactivity. Overall, it is clear that while the ability of LTF to both sequester iron and to direct reactive oxygen intermediates is a major factor in lessening damage due to excessive inflammatory responses, further effects are apparent through direct control over development of higher order immune functions that regulate pathology due to insult and injury. This culminates in attenuation of pathological damage during inflammatory injury.

## Introduction

Over the last several decades, much has been revealed about the nature and function of various immune mediators during the development of host innate immune responses to injury and infection, including cytokines, chemokines, and specific cell surface receptors which trigger a cascade of signaling pathways. There is overwhelming evidence that lactoferrin (LTF), as a first line defense mediator, plays a key role in normalization of insult-induced reactions that disrupt immune homeostasis (1, 2).

Lactoferrin is a highly conserved, monomeric 80 kDa single polypeptide chain contained in most mammalian exocrine secretions, such as milk, saliva and tears, bronchial, and intestinal secretions. LTF is also found in the secondary granules of neutrophils. The primary structure of LTF is well characterized for multiple species (3). LTF is a single polypeptide chain comprised of 692 amino acids organized in two highly homologous structured lobes, which are designated the *N*- and *C*-lobe. Each lobe is capable of binding a single ferric ion (Fe^+++^). There are two primary forms of human LTF, one is contained within exocrine secretions and the other form is located in the secondary granules of neutrophils; both forms are products of the same gene. While the secreted form is understood as involved in the host defense against microbial infection at mucosal sites, the neutrophilic LTF has additional and distinct immunomodulatory function (4). LTF is a glycoprotein, and in humans the glycans are the *N*-acetylactosaminic type, described as α1,3-fucosylation on the *N*-acetyl-glucosamine residue which is then linked to the peptide chain. In human LTF, there are three possible *N*-linked glycosylation sites; one site at Asn138, a second at Asn479, and another located at position Asn624. Differential glycosylation at these sites results in distinct variants (5). Many observed activities of LTF (both direct and indirect toward immune regulation) are dependent upon those specific and varied patterns of glycosylation (6). Although there is a high-amino acid sequence homology in species’ LTFs the unique glycosylation patterns are likely responsible for heterogeneity of their biological properties. For example, the immunoregulatory human LTF activity is dependent on the interaction of glycoprotein with a receptor specific for sialic acid, with a requirement for sialylation to permit direct lymphocyte activation (7, 8). The type of glycosylation on LTF also affects myelopoiesis (9), apoptosis (10), and netosis (11).

**Figure 1:**
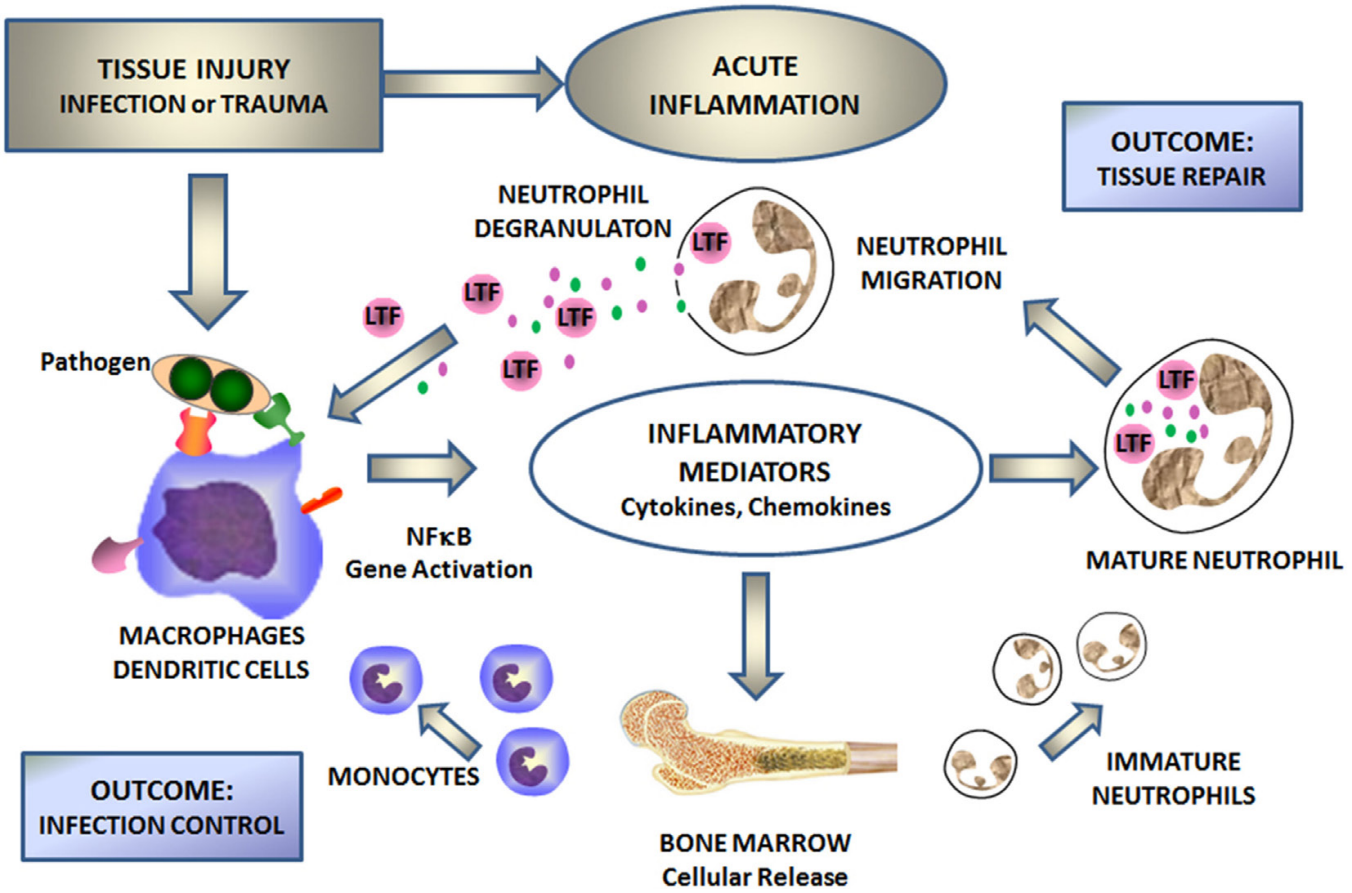
Lactoferrin (LTF) mediates cellular responses to environmental insults. Injury defined by infection, or trauma leads to activation of the NF-κB signal transduction pathway within monocyte/macrophages and/or dendritic cells. This in turn stimulates the production of inflammatory mediators, which subsequently stimulates the production of fresh neutrophils and monocytes from bone marrow and activates circulating neutrophils. Activated neutrophils degranulate to release secondary mediators, including LTF. By interacting with specific receptors on monocytes/macrophages and other immune and non-immune cells, LTF attenuates inflammation and contributes to tissue repair and limits spread of infectious agents.

Lactoferrin is a first line defense protein for protection against microbial infections (12, 13) and subsequent development of systemic disease as seen with systemic inflammatory response syndrome (SIRS) and sepsis (14–16). Clinical importance of LTF to control these processes has been clearly demonstrated through groundbreaking studies on neonates (17–19), where supplementation of diet with LTF reduced occurrence of late onset sepsis. LTF has indeed been proven as a major innate immune responder important in control of the development of acute septic inflammation (4, 14–16, 20–22). Upon infection, the monocyte/macrophage system responds with the production of inflammatory mediators, which in turn induce bone marrow for generation of new immune cells and activate degranulation of mature neutrophils (Figure 1). Subsequently, a massive amount of LTF is released from the neutrophil’s secondary granules to combat the infection. The antimicrobial activity of LTF is well documented and consists of two mechanisms: one is iron dependent and deals with high affinity of LTF to iron (bacteriostatic), and the other one is due to LTF affinity to lipopolysaccharide (LPS) to function as a direct bactericidal agent for Gram-negative organisms. Small changes, such as single nucleotide polymorphisms, can affect outcomes against pathogenic agents (23, 24). LTF interacts with cell surface receptors involved in “danger signal” recognition [e.g., toll-like receptor (TLR)4, CD14, and CD22] (8). By virtue of iron sequestration, LTF can control oxidative burst as macrophages and neutrophils initially respond to injury. In addition, by coupling with various membrane receptors, LTF affects “danger signal” activation of monocytes/macrophages. For example, LTF binding to CD14 receptor competes with the bacterial LPS (product of dying bacteria) (25) and can attenuate NF-κB-induced transcription of genes for various inflammatory mediators (26). Thus, in part, LTF is mechanistically acting as a feedback mediator for acute inflammation. Figure 1 schematically represents a sequence of events during the development of acute inflammation with LTF as a key mediator.

## Lactoferrin as a First Line Defense Mediator

In a broader sense, LTF may be classified as an acute phase protein (27) and acts as an “alarmin,” a small family of proteins released from neutrophils upon infection (28, 29), and playing an important role in altering immune reactivity upon subsequent, pathogenic encounter, or clinical insult. LTF as an alarmin establishes conditional links between neutrophils and dendritic cells (DCs) in a localized space (30). This in turn promotes the maturational switch from innate to adaptive immunity.

Moreover, LTF is a natural adjuvant enhancing immunity to all infectious agents as they are encountered during the period of infancy onward. This begins *in vivo* with mother’s milk; LTF is nature’s way to assist in the acquisition and development of protective immunity against infectious-related pathologies during early stages of ontogeny. LTF is involved in a variety of immunoregulatory functions (31–35), ranging from innate immunity enhancement to potentiation of adaptive recall responsiveness (36). It also has associated chemoprotective activity extending to multiple immune cells (37). There is sufficient evidence that LTF ameliorates insult-induced injury and systemically protects integrity of various organs during the development of inflammation (38–42). These findings are critically important in efforts to developing therapeutically relevant protocols to contain the pathological damage caused by specific diseases.

Many reviews have historically focused on LTF as an iron-binding protein involved in defense functions (43, 44), with specific analysis on its physical properties (45, 46), interaction with specific innate cell phenotypes (47, 48), or even receptor interactions that confer pathophysiology (49). For over two decades, our group has also contributed to this platform, with reviews directed toward understanding LTF’s involvement in immune modulation (1, 2). What is missing from the literature is an assessment of the direct impact of LTF on the development of inflammatory responses to alter outcomes of pathogenesis due to excessive inflammatory responses. Therefore, the objective of this monograph is to review major studies that target the functionality of LTF toward control of insult-induced inflammation and subsequent pathologies. Specific examples will highlight its utility to alter acute pathologies, allowing functional proof for global hypotheses and mechanisms of action attributed to LTF.

## Inflammation: An Immune Response to Injury

Inflammation is currently viewed as a complex pathophysiologic process that engages literally hundreds of mediators and different cell types in response to microbial or non-microbial injury. Although inflammation is important in tissue repair and/or pathogen eradication, when it is not contained in a timely manner it can be detrimental to the host by establishing systemic and often chronic inflammatory conditions. It is now apparent that the production of primary immune mediators, which include cytokines and chemokines, is dependent on the recruitment of inflammatory cells, particularly innate immune cells such as neutrophils, macrophages, and DCs. Cellular activation leads to release of secondary immune mediators and subsequent induction of the adaptive immune responses (50). In parallel, the complement system, an assembly of soluble enzymatic proteins and peptides in the blood and body fluids, actively regulates these inflammatory responses (51), many of which are identified as contributing toward regulation of higher immune function (52). The multiple interconnections among immune cells, cytokines, chemokines, and complement proteins protect against development of systemic infections and support damaged tissue repair.

Here, we review the role of LTF innate immune functions critical during the inflammatory responses which drive development of pathology. Our discussion begins with a presentation of inflammation in response to environmental insult, thus introducing entry points for LTF with the overall goal to identify mediation and subsequent control to limit pathological damage. Inflammation is a protective strategy that functions to remove a harmful stimulus, typically infectious agents. Inflammation also contributes to ultimate repair of damaged tissue, even in the absence of infection. In fact, “sterile” inflammation is a natural response elicited during tissue damage or excessive cell death (53). Acute inflammation, which occurs immediately after injury, may subsequently progress to chronic conditions if the initial response cannot be quickly resolved, either because of the persistence of the injurious agent, or due to impairment of the individual’s immune system. Typically, acute inflammation is regarded as local vasodilation and increased vessel permeability to improve blood flow and deliver molecules and cells to help repair injured parenchyma. In contrast, chronic inflammation is characterized by simultaneous or cycling destruction and subsequent healing processes over time in tissue undergoing constant and repetitive damage.

**Figure 2:**
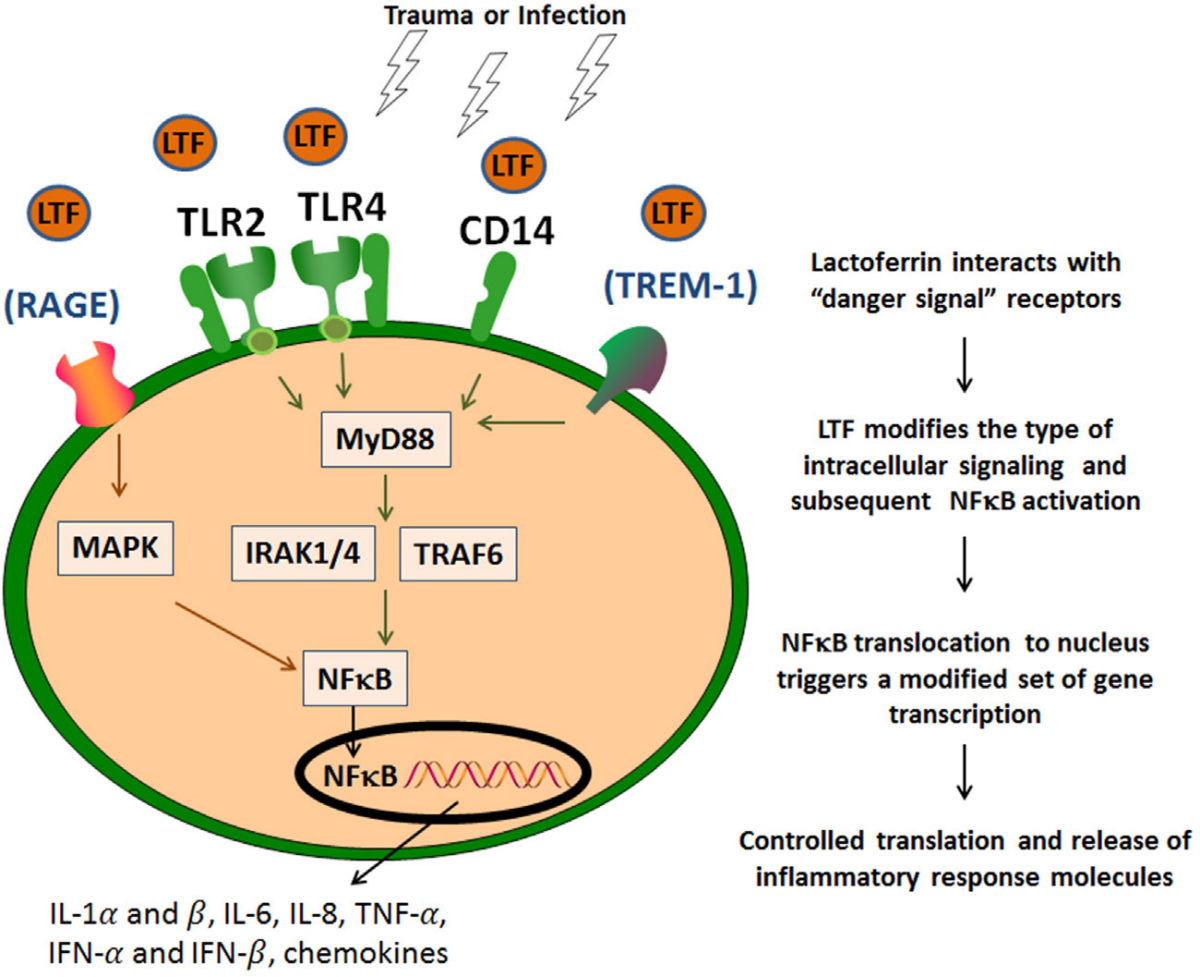
Lactoferrin (LTF) affinity to “danger signal” receptors. LTF modifies the type of intracellular signaling and subsequent NF-κB activation *via* identified danger signal receptors [toll-like receptor (TLR)2, TLR4, and CD14]. This results in a modified subset of proteins to control and contain the inflammatory response. LTF may also act *via* other additional signaling receptors, such as RAGE or TREM-1, which also utilize NF-κB activation pathways.

Both conditions of infection and sterile tissue injury generate a strong immune response induced by a sequence of events that originate from dying/injured or infected cells, resulting in release of proteins and bio-active compounds. These endogenous factors are known as cell death-associated molecular patterns or damage-associated molecular pattern (DAMP), or pathogen-associated molecular pattern molecules [reviewed elsewhere in detail (51, 52)]. These molecules are expressed by immune and non-immune cell types and are derived from their cytosol, nuclear, or mitochondrial compartments. These are exemplified by IL-1α (nucleus), ATP, S100 proteins, high-mobility group box 1, and uric acid (cytoplasm), formyl peptides, heat shock proteins (exosomes), mDNA (mitochondria), heparan sulfate, and hyaluronan fragments (extracellular matrix). These endogenous “danger signals” have the affinity for diverse cell receptors; their binding initiates coupled signaling pathways that activate inflammasome platforms and NF-κB multiprotein complexes (54) (Figure 2). LTF, like many other human milk proteins, clearly contains multiple entry points for interaction with innate receptors that mediate downstream activities related to “danger signals” (55). LTF can specifically interact with cellular receptors that sense these “danger signals,” and therefore influence innate system cells; thus serving as a mediator to migration and maturation of subsequent immune responses (48). It is readily accepted that TLRs represent a key molecular link between any type of injury and subsequent development of inflammation. This in turn leads to maturation of subsequent immune responses in a controlled manner (56). For example, LTF reduced the synthesis of chemoattractants, such as IL-8 and monocyte chemoattractant protein-1 that were induced by viral infection in macrophages, by way of suppression of NF-κB activity. LTF is perhaps best known for its interaction with accessory molecules, such as CD14, and upregulates CD40 after interaction with TLR4 (57). Furthermore, LTF has been demonstrated to neutralize free LPS by disrupting formation of complexes to activate the TLR4 signaling pathways (58). Likewise, LTF also interacts with TLR2 to interfere with molecular processes upstream of NF-κB activation. And it was reported that LTF was able to inhibit TLR9 recognition of dsDNA *via* binding to its co-receptor CD14 (59). In addition, LTF interacts with CD22 (8), and by doing so, it could reverse a methotrexate-induced suppression of the secondary immune responses of mouse splenocytes to antigen; the immunorestorative activity was dependent on the interaction of LTF with the CD169 sialoadhesin. It is therefore likely that LTF also plays an indirect role through other “sensing” receptors, such as the receptor for advanced glycation end products (RAGE) and the myeloid cell triggering receptor TREM-1. These are less prominent, but stimulation through these receptors also triggers NF-κB activation and subsequent transcription of genes for inflammatory cytokines and chemokines (60, 61). This includes expression of IL-1α and β, IL-6, IL-8, TNF-α, IL-12, IL-15, IFN-α, and IFN-β, cyclooxygenases (COX1 and COX2), and inducible nitric oxide (nitric oxide) synthase; they also include the gene products important for initiation of adaptive immune responses (such as CD80, CD86, and CD40) (62). These diverse mediators initiate a cascade of specific and non-specific responses including bone marrow stimulation for the production of monocyte and neutrophil precursors (Figure 1) (61). Finally, it should be mentioned that LTF can also activate complement, through the classical pathway, which assists in mediating attraction of innate cells to inflammatory sites (63). However, conflicting data demonstrate that LTF can block deposition of C3 and C5 complement components (64). Indeed, LTF-derived peptides have been shown to exhibit anti-classical complement pathway effects (65).

Neutrophils directly produce LTF, which upon release plays a pivotal role in the development and resolution of inflammation. LTF also increases the activity of cathepsin G, a known antibacterial enzyme (66), and broadens its substrate specificity (67), suggesting that LTF acts in concert with antibacterial neutrophil-derived enzymes in the initial phase of infection. However, it should be noted that there is a relative feedback loop; LTF released from neutrophils can effectively reduce neutrophil extracellular traps, important in overall inflammatory response (11). It is only secondary to neutrophilic release of LTF that macrophages, under conditions of inflammatory insult, are modified in responding action. LTF can modify iron homeostasis, which is critical for responsiveness to bacterial insult and subsequent inflammation (68).

### Lactoferrin Controls the Oxidative Cell Injury

By virtue of iron sequestration, LTF controls the physiological balance of production of reactive oxygen species (ROS) and rate of their elimination, which naturally buffers against direct oxidative cell injury. One strong hypothesis is that by controlling oxidative stress, LTF modulates innate immune responsiveness which alters production of immune regulatory mediators that are important for directing development of adaptive immune function. Indeed, many investigators have shown that LTF exhibits profound modulatory action on the adaptive immune system (34, 35, 44, 69), as we detail below; it is likely this stems from effects of early innate responses related to development of oxidant species. We have specifically identified that LTF has a significant regulation on cellular redox *via* upregulation of key antioxidant enzymes (70).

**Figure 3:**
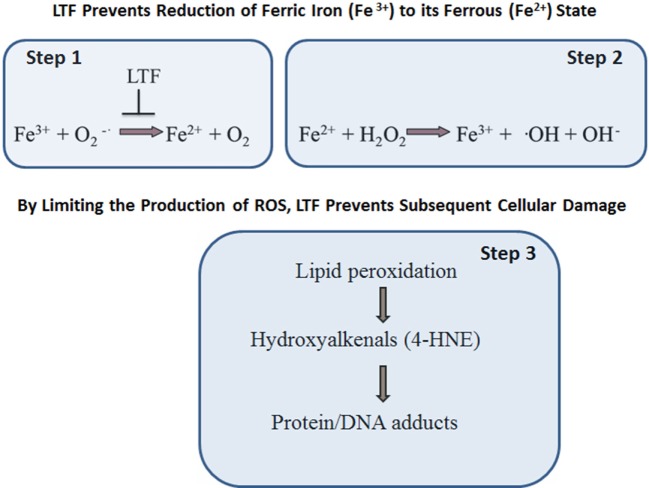
Lactoferrin (LTF) protects against oxidative stress-induced cellular damage. LTF inhibits free ferric (Fe^3+^) ions reactivity with superoxide molecules, thus limiting formation of ferrous (Fe^2+^) salt and ground state oxygen (step 1). In turn, there is reduced reactivity of ferrous (Fe^2+^) ion with hydrogen peroxide to form ferric (Fe^3+^) salt, a hydroxyl radical, and an alcohol (step 2). The end result is that LTF protects against oxidative stress, in particular by limiting the production of hydroxyl radical and lipid peroxidation (step 3).

Oxidative stress is implicated in multiple chronic degenerative processes including those which affect the development of cancer and neurodegenerative disorders, atherosclerosis, inflammation, and aging, and even defense against infection (71–73). Many of the exact oxidant species that are produced during metabolic reactions are still being researched. In many instances, the factor(s) of physiological or pathophysiological significance causing a sustained ROS production imbalance that results in subsequent establishment of oxidative stress *in vivo* remain undefined. What is known is that the rate and magnitude of ROS formation and their elimination under normal physiological conditions is highly dependent on efficiency of superoxide dismutase (SOD), catalase (CAT), and glutathione peroxidase (GPx). While SOD converts superoxide radical (•O^−2^) into hydrogen peroxide (H_2_O_2_), the other two enzymes, GPx or CAT, transform this highly reactive hydrogen peroxide into water (H_2_O) or water, and molecular oxygen (O_2_), respectively. However, in a presence of free ferric ions (Fe^3+^) the superoxide radical may go under two-step, non-enzymatic, degradation process (Figure 3). In the first reactive step, a superoxide molecule reacts with ferric ion (Fe^3+^) to form ferrous salt (Fe^2+^) and the ground state oxygen. In a second step, known as the Fenton reaction, ferrous (Fe^2+^) ions react with hydrogen peroxide to form ferric salt (Fe^3+^), a hydroxyl radical, and an alcohol. The formation of hydroxyl radical *via* an iron-dependent reaction is strongly implicated microbicidal activity within phagocytes and lipid peroxidation events (particularly polyunsaturated fatty acids). The reaction of the hydroxyl radical with polyunsaturated fatty acids results in the abstraction of a hydrogen atom. This initiates lipid peroxidation, and the production of intermediates such as hydroxyalkenals, to realize new radicals to potentially induce functional changes in multiple macromolecules of biological importance. These macromolecules include DNA, proteins, and lipids (74). By virtue of sequestering ferric (Fe^3+^) ions, LTF protects against the damaging effects of oxidative stress.

Lactoferrin contributes to general homeostasis by disrupting the production of these metabolically active molecules (70). This has been demonstrated in models of tissue trauma. Okazaki et al. (26) investigated the antioxidant property of bovine LTF (bLTF) in the rat model of ferric nitrilotriacetate-induced renal tubular oxidative injury. In this study, LTF suppressed both urea nitrogen levels in the blood and elevation of serum creatinine. Protective effects against renal oxidative tubular damage were seen, as well as maintenance of antioxidant enzyme activities in the bLTF-pretreated group. The authors suggested that LTF intake is beneficial for the prevention of iron-mediated renal tubular oxidative damage.

Also, in earlier studies on endotoxemic mice, it was demonstrated that LTF decreased levels of intracellular oxidative stress induced by LPS through reduction in mitochondrial dysfunction (75). In that particular study, LTF attenuated mitochondrial dysfunction within the liver of LPS-treated animals. This was demonstrated by reduced release of H_2_O_2_ from mitochondria, as well as a significantly lowered associated mitochondrial DNA damage. This supports a growing body of evidence that LTF can modulate cellular damage and death induced by acute inflammation. Apoptotic and necrotic cell death are two consequential activities critical to development of SIRS and sepsis-related pathology. These are tightly associated with mitochondrial dysfunction that is often characterized by increased production of ROS and increased membrane permeability, loss of mitochondrion integrity, and alterations in levels cellular ATP. Mitochondrial dysfunction and attendant bioenergetics defects are indeed increasingly becoming recognized as important role players in many chronic and acute disorders (75).

## Lactoferrin and General Physiologic Homeostasis

Homeostasis is the maintenance of equilibrium in a biological system by means of feedback *via* control mechanisms which counteract physiological dissonance and/or development of pathology. At the molecular level, homeostasis is controlled by a neuro-endocrine-immune system network wherein LTF plays a central role, largely due to its above-mentioned ability to bind ferric ions. Iron indeed is the most abundant trace mineral found within the body and it plays an essential role in most biological systems. Iron is a vital component of the heme in myoglobin, in hemoglobin, and in cytochromes. Iron is also an essential cofactor for many non-heme enzymes, including ribonucleotide reductase which is a limiting enzyme for DNA synthesis (76). However, free iron is also toxic in that it has a strong potential to induce formation of “dangerous” free radicals. Consequently, iron homeostasis is tightly regulated, in part by LTF, which plays a role of “safety net” protecting against oxidative stress to reduce the magnitude and the structure of insult-induced cellular damage.

### Lactoferrin As a Sensor of Immune Status in Humans

**Figure 4:**
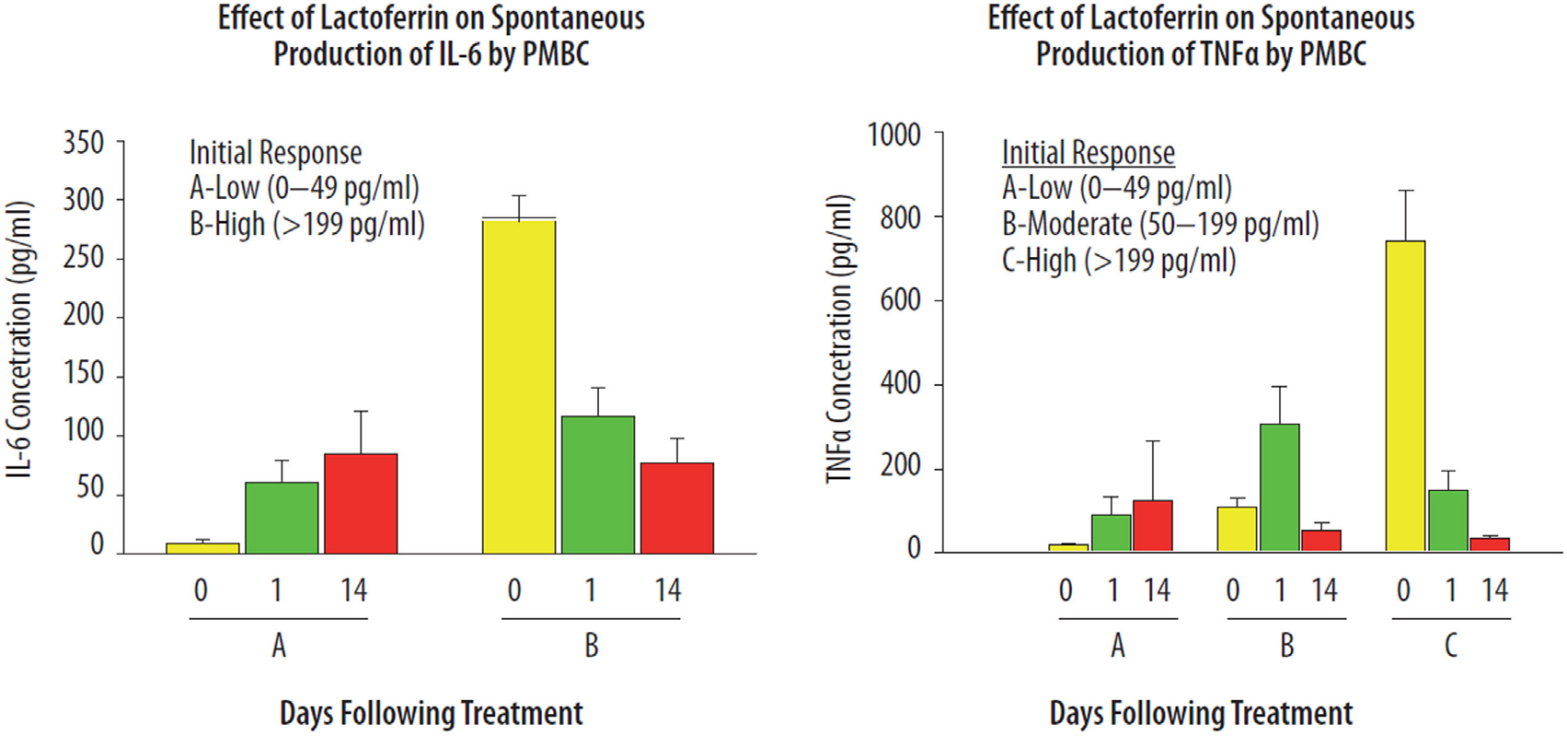
Immunoregulatory effects of lactoferrin (LTF) on the spontaneous production of IL-6 and TNF-α from human PMBCs. Healthy volunteers were given one capsule containing 10 mg of LTF, daily for 7 days. Blood samples were examined prior to the first dose, and again 1 and 14 days after administration of the last dose of LTF. The spontaneous production of IL-6 (left) and TNF-α (right) from blood cells was determined by bioassay after 24 h whole blood culture incubation. The data presented have been published previously, in Kruzel et al. (2), reprinted with permission.

Lactoferrin is a pleiotropic agent involved as an *in vivo* “immune sensor” directing specific immune responses to acquire immune homeostasis (2). However, one of the key questions emerging from studying the pleiotropic immune activities of LTF *in vivo* is a determination of factors that drive LTF to exhibit directed anti- or pro-inflammatory parameters. In a human volunteer study, orally administered bLTF (40 mg/day) showed differential effects on immune responsiveness of peripheral blood mononuclear cells (PBMC) (2). Healthy individuals were evaluated for the effects of LTF on the mitogenic proliferative response of PBMCs, and the ability of whole blood cultures to produce IL-6 and TNF-α upon LPS activation. Two categories of individuals were identified (high and low responders) based on immune responsiveness of PBMCs to secrete IL-6 (Figure 4). Another group was selected according to relative production of TNF-α. The *in vivo* effects of LTF appeared to be dependent upon the baseline immune status of a given individual. Down-regulatory activity of LTF was identified after treatment in the high-responding individuals, while the lower-responding group showed that LTF had upregulatory effects. To a much greater extent, LTF affected spontaneous production of TNF-α and IL-6 by PBMCs. It is known that cytokine production is dependent on interaction *via* cell-to-cell receptors (77), which include the accessory adhesion molecules such as LFA-1. Indeed, experimental models demonstrated that LTF has regulatory effects on cytokine production that is linked to modulation of LFA-1 surface expression (35). A third parameter was identified in which a twofold increase in the content of neutrophil precursors in the circulating blood was noticed after oral treatment of volunteers with LTF was (not shown). It has been suggested that the dichotic responses seen in individuals are related to genetic polymorphisms present within the population (24, 78–80).

The immunoregulatory and protective activities of LTF were subsequently established in mice (81) and patients (82) subjected to surgery. In the first case, LTF significantly reduced levels of TNF-α and IL-6, elicited by abdominal surgery. In the patients, in turn, LTF prevented postsurgical hyporeactivity of peripheral blood lymphocytes, typically observed 1 day following surgery. Intraperitoneal and intravenous routes of LTF administration ensure rapid and undisturbed interaction of target cells with undigested LTF. With that in mind, the numerous immunoregulatory effects of LTF when given orally or buccally require particular attention and a plausible mechanistic explanation. It appears that LTF has the potential to interact with several cell types contained in oral mucosa. Epithelial DCs (83), keratinocytes (84), and oral epithelial cells (85) bear CD14 and TLRs able to recognize LTF. More importantly, LTF can trigger maturation of DCs through TLR2 and TLR4 (86), with such events known to promote DC migration to adjacent lymph nodes (87). Orally ingested LTF may also interact with intestinal epithelial cells expressing TLRs (88). Thus, the immunoregulatory effects of oral LTF may originate from its interactions with target cells located in the sublingual and buccal regions of the oral cavity. Most probably, LTF bearing DAMP characteristics transmits “danger” signals by DC into peripheral lymphoid tissue. LTF may not only transmit direct signals upon interaction with mucosal cells, but may also penetrate target organs (55, 89), such as seen after high-systemic oxidative stress (90). However, further studies are required to explain how these DC communicate with other immunocompetent cells to change their activation status. In a more recent study, we evaluated effects of homologous, recombinant human LTF (rhLTF) on the reactivity of whole blood cells derived from patients admitted to the ICU because of surgical, medical, or trauma reasons (91). Some of the patients developed septic shock. The effect of LTF was investigated with regard to LPS-induced TNF-α production following admission to ICU. The patients showed, in majority, a deep hyporeactivity to LPS upon admission. The rhLTF exerted differential effects on production of LPS-induced TNF-α in those patient’s whole blood cell cultures. The cytokine production was upregulated only in patients with sustained anergy to LPS, and inhibited or unchanged in moderately reactive patients.

### Lactoferrin Protects Intestinal Integrity during Endotoxemia

For many years, it has been known that LTF has protective action to limit irritation within subcutaneous tissues and internal organs. For example, oral treatment of rats with bLTF inhibited carrageenan-induced paw inflammation in rats (41). LTF inhibition of the skin inflammation was associated with a profound decrease in the ability to produce LPS-induced IL-6.

**Figure 5:**
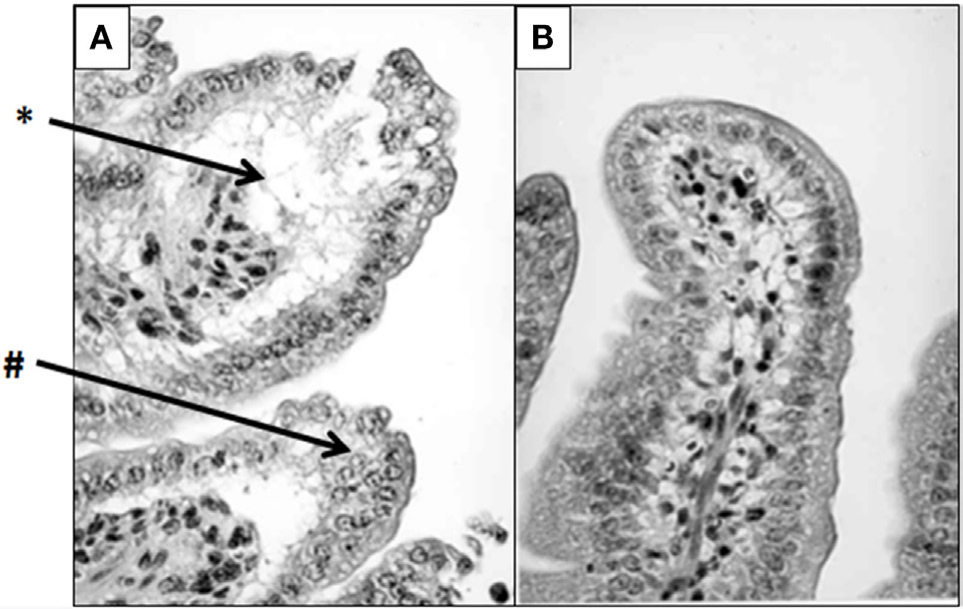
Lactoferrin (LTF) protects against lipopolysaccharide (LPS)-induced damage to mouse intestinal structures. LTF administered intraperitoneally prior to injection of LPS significantly limited damage to intestinal microvilli. High-power micrographs reveal that the saline/LPS-treated mice **(A)** demonstrate severe villus atrophy (#), edema, and epithelial vacuolation (*) compared with the LTF-treated counterparts **(B)**. Tissue was stained with hematoxylin and eosin as described in Ref. (1). Photomicrographs were previously described in detail, and published in Actor et al. (1). Visualized at 400× magnification.

There is expanding evidence to show that progression of SIRS into sepsis is due to cellular damage caused by acute inflammatory responses (92, 93). The cell death during these events is in part due to mitochondrial dysfunction, often characterized by increased production of ROS (94). LTF is a critical component involved in mediation of this response, so as to allow controlled regulation of inflammation without rapid induction of pathological damage. The mechanism of action for LTF contains multiple components for differential regulation of cellular immune responses during the development of SIRS. Bacteremia and endotoxemia manifest as severe clinical syndromes characterized by pro-inflammatory cytokine release, massive release of ROS, and increased adhesion molecule expression (95, 96). In particular, the systemic inflammatory response directed toward bacterial LPS includes production and liberation of pro-inflammatory cytokines from gut-associated lymphoid tissue, which in turn affects gut mucosal permeability. This subsequently contributes to enteric bacterial translocation to distant sites (97–100). As demonstrated in mice, intraperitoneal administration of LTF at 1 h prior to LPS injection significantly enhanced survival and reduced mortality from 83.3 to 16.7% (20). Similar protective effects have been reported by Zagulski et al. using live *Escherichiacoli* (101). Histological examination of intestinal segments revealed remarkable differences between LTF-pretreated endotoxemic mice and LPS counterpart. Severe vacuolar degeneration of jejunal epithelium observed in LPS-treated mice was significantly reduced in mice treated with LTF [Figure 5; previously published and described in detail by Actor et al. (1)]. Clearly, LTF has an impact on gut mucosal integrity and function (102). Mice treated with LPS quickly become hypothermic; pretreatment with LTF allowed a less pronounced drop in body temperature. Development of hypothermia in those LPS-treated mice also correlated with severe lethargy; LTF-treated mice expressed normal behavioral activities. At the molecular level, LTF seems to reduce LPS-induced monocyte activation and subsequent production of pro-inflammatory mediators. Similarly, rats given LTF to modulate LPS treatment showed marked moderation of hypotension and alleviation of histopathological damage to intestinal tissue (103). Proof of concept models using *E. coli* and/or MRSA infection have been detailed (1, 104–106); administration of LTF resulted in uniform inhibition of TNF-α, IL-6, and IL-10, as well as reduction in NO. Of high interest, prophylactic administration of LTF resulted in similar decreases in both TNF-α and NO. Similarly, LTF given 18 h postinduction of endotoxic shock gave nearly identical results (1). The mechanisms of action for LTF to protect gut integrity appear to contain multiple components for differential regulation of cellular immune function during *in vivo* models of sepsis in both mice and rats. In fact, a strong resulting hypothesis was put forth that the protective effects of LTF result from generalized deactivation of the macrophage population, which is manifested by significant suppression of both pro- and anti-inflammatory mediators (1, 16, 20).

Similar pathological changes in organs, associated with inadequate inactivation of intestinal LPS, have been reported by ligation of bile duct (107). A complete deprivation of bile (s-IgA and containing bile salts) from the gut lumen leads to loss of mucosal integrity, and decreased endotoxin inactivation in the endogenous bacterial flora. The result is portal bacteremia and endotoxemia, as well as increased bacterial translocation to mesenteric lymph nodes. In an experimental model, we evaluated effects of bLTF, on liver histopathology in obstructive jaundiced (OJ) rats and the production of LPS-induced TNF-α and IL-6 by splenocytes (108). Table 1 summarizes the effect of LTF on the intensity of induced OJ. In rats with 14-day OJ, treated with bLTF, the changes in the liver pathology were markedly reduced in multiple parameters that included limitation of necrotic foci with disseminated lymphocytes, and reduced cellular necrobiosis and bile duct proliferation and dilation. Concurrent with this was an absence of proliferation of fibrous and reticular connective tissue or Kupffer cell activation. In these rats, LTF upregulated cytokine production, in particular spontaneous and LPS-induced TNF-α indicated a reversal of LPS hyporeactivity caused by long-time endotoxin exposure. Innate immunity parameters in the small intestine may be upregulated by LTF, shown in mice subjected to immobilization stress (109). Other studies were devoted to evaluate protective effects of bLTF on colitis induced by application of non-specific chemical irritants. In a model of trinitrobenzenesulfonic acid (TNBS)-induced colitis in rats (110), daily gavage of LTF attenuated TNBS-induced colitis as assessed by macroscopic and histologic scoring and myeloperoxidase activity. The anti-inflammatory cytokines IL-4 and IL-10 were upregulated whereas pro-inflammatory TNF-α and IL-1β were suppressed. Similar results were obtained in a model of dextran-sulfate-induced colitis in BALB/c mice (111). In this study, the treatment of mice with apo-LTF (iron-free) gave better results than that with holo-LTF (iron saturated). Very recent findings also indicate that in the model of dextran sodium sulfate induced colitis, LTF protects gut integrity by increasing T regulatory cell population in intestinal lamina propria (112).

**Table 1:** Type and degree of changes in livers of rats with obstructive jaundice.

Type of change	Treatment	Mean	Median	Statistics	No change	Light change	Moderate change	Significant change	Total
Foci with necrosis with disseminated lymphocytes	Control	2.29	9.73	*p* ≤ 0.001	0	2	6	6	32
	LTF	0.17	0.39		10	2	0	0	2
Necrocytosis of single hepatocytes	Control	1.29	0.61	*p* ≤ 0.001	0	11	2	1	18
	LTF	0.25	0.45		9	3	0	0	3
Proliferation of the bile ducts with their dilations	Control	2.29	0.73	*p* ≤ 0.001	0	2	6	6	32
	LTF	0.58	0.51		5	7	0	0	7
Proliferation of fibrous and reticular connective tissue	Control	1.00	0.00	*p* ≤ 0.001	0	14	0	0	14
	LTF	–	–		12	0	0	0	0

*Changes seen in liver tissue samples were scored as none, light, moderate, and significant, as described (108). The non-parametric Mann–Whitney’s test was applied to data, with results given as mean and median values. Significant differences are indicated; *n* ≥ 12 per group. Data previously published by Zimecki et al. (108)*.

Intestinal ischemia–reperfusion injury (I/R) is a serious clinical condition, also affecting gut integrity. A study examined LTF for its properties to attenuate I/R in rats (113). Under non-treated conditions, I/R is reflected by morphological alteration, reduction of γ-glutamyl transpeptidase (γ-GGT) activity, and increased cell apoptosis. In these studies, I/R damage was achieved by an occlusion of the superior mesenteric artery. Daily administration of LTF for 14 days before surgical intervention significantly attenuated gut damage reduced the histologic score and apoptosis index. LTF treatment restored intestinal γ-GGT activity, reduced intestinal malondialdehyde, and myeloperoxidase levels, reestablished glutathione levels, and lowered serum levels of pro-inflammatory cytokines. Orally ingested bLTF also protected intestinal damage elicited by experimentally induced hepatitis (39).

Among many therapeutic applications of LTF in humans, bLTF, ingested daily for a period of 1 year in a high dose (3 g), suppressed colorectal polyps growth in patients (114). The patients with regressing polyps had increased NK cell activity and increased autologous LTF serum levels. The polyps showed the presence of CD4^+^ and CD161^+^ cells, suggesting that infiltrating T and NK cells play a role in tumor destruction. Taken together, LTF exhibits protective functions in jejunum, colon, and liver of experimental animals exposed to bacterial products or chemical irritants by means of several mechanisms, all of which appear related to control of ROS.

### Lactoferrin Ameliorates Pollen Antigen-Induced Airway Inflammation

**Figure 6:**
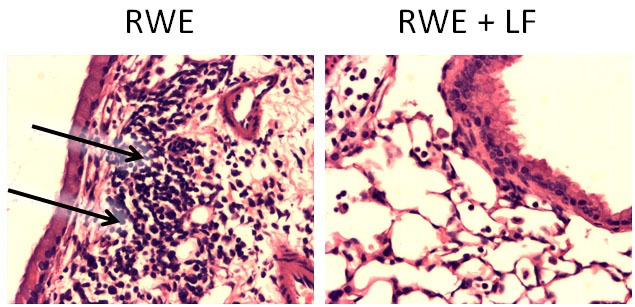
Effect of lactoferrin (LTF) on ragweed pollen (RWE)-induced accumulation of inflammatory cells into subepithelium. Mice challenged with RWE demonstrated high-inflammatory cell infiltrates within peribronchial region (arrows) postchallenge (left), but not in the LTF-treated mice (right). Sections were stained with hematoxylin and eosin and visualized at 100× magnification. Photomicrographs were previously described in detail, and represent studies published in Kruzel et al. (38).

Oxidative stress in asthma is a consequence of enhanced ROS generation by inflammatory cells recruited to airways upon concurrent exposure to pro-oxidant environmental molecules (ozone, cigarette smoke, etc.), or to respiratory viral infections (115, 116). We have previously shown that pollen grains and their extracts, such as ragweed pollen (RWE), generate ${\text{O}}_{2}^{\cdot -}$ because of reduced NAD(P)H oxidase activity, essential for robust airway inflammation generated in the mouse (117, 118). Although ${\text{O}}_{2}^{\cdot -}$ and H_2_O_2_ are considered as signaling molecules (119), their conversion to ^•^OH by free iron is important in induction and augmentation of inflammatory processes. Pro-oxidant iron is present in airway lining fluids, as well as in resident cells of airways of human and animals. This pro-oxidant iron mediates oxidative stress in the lungs (120–123). LTF can certainly modify iron homeostasis, critical for responsiveness to bacterial insult, and subsequent inflammation, as discussed previously (68). We demonstrated that LTF, as an iron-binding protein, ameliorated (RWE)-induced airway inflammation. Interestingly, only apo-LTF (iron free), but not the iron-saturated holo-LTF form significantly lowered accumulation of inflammatory cells and formation of mucin-producing cells in airways induced by pollen extract [Figure 6; previously published and described in detail by Kruzel et al. (38)].

The utility of LTF to limit and contain ovalbumin-induced pleurisy in BALB/c mice was studied (124). Pulmonary histologic analysis showed that LTF diminished pathological lesion development, including diffuse alveolar hemorrhage and hemosiderosis and accumulation of pulmonary edema, which were common in control lungs after injection of the eliciting dose of ovalbumin. LTF also decreased the amounts of IL-5 found secreted into pleural fluid, suggesting a skewing of immune reactivity to T_H_1 type. This was a first demonstration that LTF significantly decreased antigen-specific pleurisy in a sensitized mouse model. The finding findings were confirmed and extended by others who showed increases in IFNγ but decreases of IL-5, 10, 17, and TGFβ1 production which are typical markers for T_H_2 and T regulatory cells function and development (125). An alternate explanation includes the ability of LTF to inhibit eosinophil migration during allergic rhinitis and asthma (126).

### Lactoferrin Ameliorates Tuberculosis (TB)-Induced Lung Pathology

**Figure 7:**
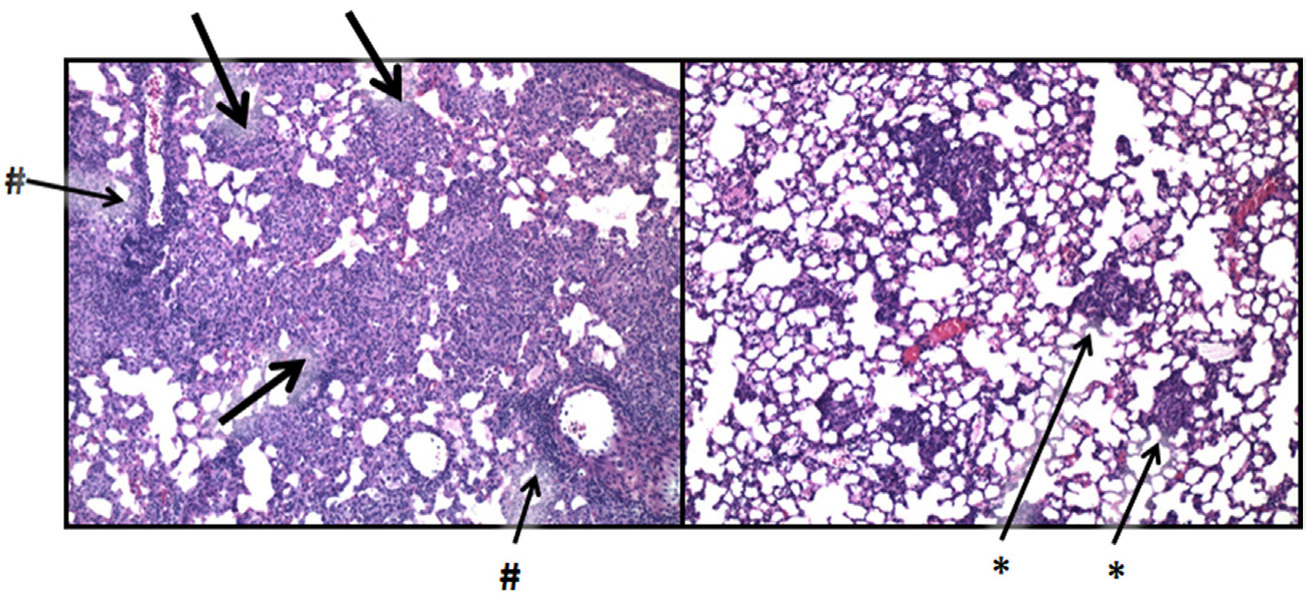
Lactoferrin (LTF) modulation of the mycobacterial granulomatous response. C57BL/6 mice were challenged with mycobacterial glycolipid trehalose 6,6′-dimycolate (TDM) prepared as described (42). TDM induces severe granulomatous responses comprised of activated monocyte/macrophages, with peak inflammatory response shown postinjection (left). Aggressive granulomatous response includes large mononuclear cell accumulation (thick arrows) and severe lymphocytic perivascular cuffing (#). Administration of LTF at 1 day postchallenge nearly abolishes the pro-inflammatory response, and restores normal lung architecture (right), with only focal residual monocytic infiltrates remaining (*). Photomicrographs were previously described in detail, and represent studies published in full in Welsh et al. (42). Similar results were described using recombinant human LTF (127). Sections were visualized at 100× magnification, stained with hematoxylin and eosin.

Lactoferrin has been shown to have therapeutic function to alter destructive pathology in models of TB. Specifically, LTF was able to modulate granulomatous responses, without significant loss of production of known early induced pro-inflammatory mediators (TNF-α, IL-1β, and IL-6) requisite for control of infectious organisms. Treatment with LTF showed statistically significant fewer granulomas compared with mice given mycobacterial cord factor (glycolipid trehalose 6,6′-dimycolate; TDM) alone (42), and granulomas were significantly reduced in size and complexity. The utility of oral delivered LTF was also demonstrated with statistically significant reduction in histopathology post-TDM administration in BALB/c and C57Bl/6 mice [Figure 7; representative data shown only for C57Bl/6 mice; data previously published by Welsh et al. (42)]. The oral delivered LTF demonstrated biologically relevant reduction in pathology, similar to that seen in the published results using intravenous administered rhLTF (127).

We have previously reported that classical cytokines critical for control of *Mycobacterium tuberculosis* (MTB), namely TNF-α, IL-6, and IFN-γ, were not statistically altered by LTF treatment (41). In one experiment, we gave LTF administered in drinking water to mice aerosol infected with virulent mycobacteria (41). The bacterial load in tissue was only slightly reduced; however, the major change was amelioration of granulomatous severity. It is noteworthy that this activity was accompanied by an increase in classical pro-inflammatory mediators while decreasing overall lung immune-histopathology (41). Specifically, the LTF-treated animals increased presence of helper CD4^+^ IFN-γ^+^ and IL-17 producing cells in lung tissue. The LTF by itself was not bactericidal, instead, the LTF was shown to augment IFN-γ mediated macrophages killing of MTB by in an NO-dependent manner. As a clinical outcome, these studies suggest strongly that LTF can be a novel therapeutic for TB treatment. The limitation of pathology and tissue damage postinfection would in essence create a “firebreak” to slow transmission within a community. The data presented in those studies indicates that LTF protects against excessive pathological changes and should be considered when discussing novel therapeutic protocols for the treatment of TB.

### Lactoferrin Mediates Differentiation of Adaptive Immune Function

It is becoming increasingly clear that LTF plays a role central to the development and full expression of adaptive host immune responses. LTF has demonstrated profound modulatory activity on the adaptive immune system (34, 35, 69) by promoting maturation of T_H_0 cells (T-cell precursors) into competent helper cells. LTF was also able to accelerate differentiation of immature B cells into efficient antigen presenting cells (128). In another experimental model, LTF bound to monophosphoryl lipid A was shown to be equally effective in augmenting antibody response to bacteria as Freund’s complete adjuvant (CFA) (129). Such a complex had negligible pro-inflammatory properties when compared with CFA, so a potential application of such an adjuvant would be conceivable in humans. Studies from our laboratory demonstrate that both bovine and human LTF added to the BCG vaccine led to increased protection in C57BL/6 mice against subsequent challenge with virulent mycobacterial organisms, shown *via* decrease in bacterial burden in the primary lung and secondary disseminated organs, such as the spleen (36, 130–132). The C57BL/6 mice vaccinated with BCG and LTF had increased expression of IFNγ mRNA within the lung soon postchallenge with virulent MTB, suggesting localization of increased T_H_1 responses at critical sites of clinical importance. The recall responses to heat-killed BCG in animals given the BCG LTF vaccine combination demonstrated increased levels of IFN-γ, in addition to other pro-inflammatory mediators, compared with the mice vaccinated only with BCG. IL-4 and IL-10 were also reduced in the BCG LTF vaccinated groups (130). Lung histopathology showed significantly reduced pathological damage in mice immunized with BCG and LTF, where focal, lymphocytic, granulomas were seen surrounded by normal lung parenchyma. These enhanced pathological protective responses induced by the BCG LTF combination vaccine extend to BALB/c mice, which typically demonstrate decreased T_H_1 responses to MTB compared with the more responsive C57BL/6 strain (133).

### Lactoferrin Inhibits Pathological Development during Autoimmune Disease

Some time ago, we showed that New Zealand Black mice which had been treated for a prolonged period with bLTF exhibited a decreased frequency of positive Coombs’ reactivity (134). Incubation of peritoneal cells with LTF resulted in decreased numbers of cells recognizing Hb erythrocyte hidden antigen on autologous erythrocytes. Since addition of PGE_1_ to the cultures of peritoneal exudate cells inhibited the number of CD5^+^ autoantibody-producing B cells by 50% (135), the stimulation of COX1 expression by LTF (49) may well explain that protective effect of LTF. In another study, oral administration of LTF was examined for changes in experimental autoimmune encephalomyelitis (EAE) in Lewis rats (136). LTF treatment led to significant acceleration of recovery, taking into account the clinical score. In addition, there was highly elevated cell numbers in inguinal lymph nodes in the EAE rats which was normalized by the LTF treatments. Furthermore, LTF decreased the elevation of serum TNF-α and TGFβ concentrations. The histological analysis of the spinal cord revealed reduction in the number and size of inflammatory foci in LTF-treated rats. In addition, a small clinical trial on subjects suffering from multiple sclerosis (137) demonstrated that oral treatment of the patients with LTF resulted in a profound decrease of IFN γ and increase of IL-10 secretion by whole blood cell cultures stimulated with cytokine inducers. These changes were accompanied with an improvement of their clinical status. The protective properties of LTF in the neurodegenerative diseases were in part explained by improving viability of Schwann cells (138). In addition, an immune deviation phenomenon may occur in this case as LTF was previously shown to preferentially inhibit antigen-specific proliferation and phenotype of T_H_1-type cell lines (139). Other studies have also concluded that skewing of adaptive T-cell function by LTF is a potential way to direct adaptive function (125, 133, 140).

Other models identifying LTF to control pathological inflammation have been detailed, many of which have described directed changes on human clinical outcomes of disease states. Yin et al. revealed a profound hepatoprotective effect due to LTF within a mouse model of hepatitis induced *via* concanavalin A administration (141), which mimics the pathophysiology of human viral and autoimmune hepatitis. LTF had protective effects attributed to its inhibition of T-cell activation and production of IFNγ, as well as a noticed suppression of IL-4 by hepatic natural killer T cells. The effects of human native LTF on experimental, caerulein-induced acute pancreatitis in rats were also studied (142). LTF significantly reduced elevation of serum amylase, and of pancreatic wet weight. Histologic alterations within the pancreas were also greatly suppressed. In addition, LTF exhibited regulatory effects on bone cell activity (143), and a profound, regenerative effect on bone structure in ovariectomized rats (144). Also, a protective effect of LTF against acute acid reflux-induced esophageal damage was demonstrated in rats (145). A selected list of LTF protective actions in various pathological states is presented in Table 2.

**Table 2:** Selected examples of lactoferrin (LTF) protective activity against defined inflammation-induced pathologies.

Experimental model	LTF activity	Immune outcome	Species	Reference
Contact sensitivity to oxazolone	Suppression of the inflammatory parameters by topical application	Inhibition of T_H_1-type antigen-specific cells activity	Mouse	(140)
Endotoxemia	Protection of intestinal integrity, reduction of mortality and morbidity, suppression of serum cytokine levels	Inhibition of ROS, deactivation of RES	Mouse	(16, 20, 146)
Gut mucosa damage by chemical irritants	Decreased histological scores	Inhibition of MPO activity and expression of IL-1β and TNF-α	Mouse	(111, 112)
Ovalbumin-induced pleurisy	Amelioration of lung pathology	Immune deviation to T_H_1 cytokine profile	Mouse	(124)
Pollen antigen-induced airway inflammation	Attenuation of lung pathology	Inhibition of ROS production	Mouse	(118)
TB-induced lung pathology	Decreased lung histopathology and bacterial burden in lung postinfection, lessened organism dissemination, increased Thelper1 response	Increased antigen presentation, modulation of cytokine environment responsible for granulomatous response	Mouse	(36, 132, 147, 148)
Autoimmunity	Extension of life span, decline in anti-erythrocyte autoantibody titer	Stimulation of COX1 expression, reduction of IL-2R on autoimmune CD5^+^ B cells	Mouse	(134)
Postoperative response (thymectomy, splenectomy)	Significant inhibition of serum TNF-α and IL-6	Desensitization of RES	Mouse	(81)
Concanavalin A-induced hepatitis	Reduction of pathological changes	Inhibition of IFN γ production by T cells and IL-4 by hepatic NK T cells	Mouse	(141)
Intestinal inflammation in experimental hepatitis	Improvement of survival rate	Enhanced expression of IL-11 and bone morphogenetic protein 2	Mouse	(39)
Renal oxidative damage	Suppression of serum creatinine and blood nitrogen urea levels	Preservation of antioxidant enzyme activities	Rat	(26)
Bone structure in ovariectomized	Increased bone mass, trabecular number and thickness, and mineral density	Regulation of OPG/RANKL/RANK pathway	Rat	(144)
Experimental autoimmune encephalomyelitis	Attenuation of inflammatory changes in the spinal cord, improvement of clinical scores	Immune deviation to T_H_2 type	Rat	(136)
Obstructive jaundice	Reduction of liver pathology	Inactivation (binding) of intestinal LPS by LTF	Rat	(108)
Intestinal ischemia-reperfusion injury	Attenuation of gut damage (histologic score and apoptotic index), restoration of γ-GGT, decrease of serum TNF-α, IL-1β, and IL-6	Antioxidative, anti-inflammatory, antiapoptotic	Rat	(113)
Colorectal polyps	Suppression of polyp growth	Increased numbers of CD4^+^ and CD161^+^ cells in the polyps and neutrophil activity	Human	(114)
Multiple sclerosis	Regulation of cytokine profile, improvement of clinical state	Immune deviation to T_H_2 type	Human	(137)
Postoperative response to minor surgery	Optimalization of LPS-induced IL-6 and TNF-α production in whole blood cultures, upregulation of PHA-induced PMBC proliferation	Correction of postoperative shock immune hyporeactivity	Human	(82)
Immunoreactivity of septic patients	Regulation of LPS-induced TNF-α production from whole blood culture	Abolishment of hyporesponsiveness of blood leukocytes to LPS	Human	(91)

Lastly, it is worth mentioning the adjunct effect of LTF in treatment of certain infections with antibiotics (149–152). The utility of LTF to control bacterial and viral growth (153–155), and onset of subsequent inflammation and tissue damage, contributes greatly to its general effects to control development of SIRS and septic pathology (16, 156). This phenomena, and similar interactions of LTF with pathogenic organisms, have been well documented in multiple excellent separate reviews (43, 46).

## Conclusion

In conclusion, LTF plays a major functional role in physiologic homeostasis as related to development of disease and associated pathology. In many cases, LTF fulfills its anti-inflammatory roles *via* different cell receptors and activation of various cell signaling pathways, often through iron-dependent mechanisms. In fact, the ability of LTF to both sequester iron and to direct reactive oxygen intermediates is a major factor in lessening damage due to excessive inflammatory responses. The immunomodulatory nature of this protein derives from its unique ability to sense the immune activation status of an organism and act accordingly. The interaction of LTF with its receptors can trigger “redundant” protective effects as reflected by (1) regulation of enzyme activities and ROS production; (2) immune deviation and modulation; (3) change of cell phenotype and cytokine profile; (4) binding to LPS or competition with its receptors, and (5) prevention of cell apoptosis. Many additional immune pathways are also affected, which culminate in the consequence of attenuated pathological changes as tissue repair processes are initiated.

## Author Contributions

MK, MZ, and JA contributed equally to the writing of the manuscript.

## Conflict of Interest Statement

The authors declare that the research was conducted in the absence of any commercial or financial relationships that could be construed as a potential conflict of interest.
